# SELF-RATED ORAL HEALTH SATISFACTION AMONG OLDER ADULTS RECEIVING ONE HOME AND COMMUNITY-BASED SERVICE

**DOI:** 10.1093/geroni/igac059.1985

**Published:** 2022-12-20

**Authors:** Steffany Chamut, Shahdokht Boroumand

**Affiliations:** Harvard School od Dental Medicine, Boston, Massachusetts, United States; National Institutes of Health (NIH), Bethesda, Maryland, United States

## Abstract

**Objective:**

To assess self-rated oral health (OH) satisfaction among recipients of the home and community-based services (HCBS) administered by the Administration on Aging.

**Methods:**

This cross-sectional study is representative of the national population of the 2014 National Survey of Older Americans Act Participants (NSOAAP), and analyzed baseline OH data from community-dwelling adults (n= 3,995) age 60 and older covering case-management, congregate-meals, home-delivered-meals, homemaker-services, and transportation.

**Results:**

Among the 3,995 participants, 35% received only one HCBS, the majority being age 75-84 (42%), females (67%), Non-Hispanic Whites (72%), and living in urban areas (53%). About 54% reported having a dental visit in the last twelve months, 30% were not satisfied with their OH, and 45% reported having 4-6 medical conditions. Congregate-meals (67%), home-delivered-meals (23%), and transportation (6%) were the most services provided (n=1,130). Being not satisfied with general health (OR=4.44, CI95=2.81-7.00) p<.0001, experiencing difficulties with three or more activities of daily living (ADLs) (OR=2.06, CI95=1.15-3.70) p= 0.0149, and not having a dental visit in the last twelve months (OR=1.66, CI95=1.06-2.59) p= 0.0248 were the strongest indicators negatively associated for not being satisfied with OH among HCBS participants.

**Conclusion:**

Oral diseases and oral microbiota are known to be precursors of dementia, Alzheimer’s, multiple systemic diseases (i.e. diabetes), and mortality. Understanding OH issues among HCBS recipients could refine policies that focus on improving functional status with person-centered services, and help identify opportunities to revamp older adults’ oral and systemic health, promote healthy aging and longevity allowing them to continue living independently at home.

